# 735. Contribution of the Oral Cavity and Gastrointestinal Microbiome on Bloodstream Infections inLeukemia Patients

**DOI:** 10.1093/ofid/ofac492.026

**Published:** 2022-12-15

**Authors:** Stephanie McMahon, B S Microbiology, Jessica Galloway-Peña, Pranoti V Sahasrabhojane, Samuel A Shelburne

**Affiliations:** Texas A&M University, College Station, TX; Texas A&M Universiy, College Station, Texas; MD Anderson, Houston, Texas; MD Anderson, Houston, Texas

## Abstract

**Background:**

Blood stream infections (BSI) are a common cause of morbidity and mortality in immunocompromised patients. It was previously reported that intestinal domination (defined as occupation of ≥ 30% of the microbiota by a single bacterial taxon) by

*Enterococcus* and Proteobacteria often preceded enterococcal and gram-negative BSI.

**Methods:**

16s rRNA sequencing was used to analyze the saliva and stool of 73 acute leukemia patients with BSIs to determine the correlation between the taxa present ( > 0% relative abundance), colonizing ( > 3%) or dominating ( > 30%) a patient’s microbiome and the BSI agent. Species level confirmation of these findings was performed via digital droplet PCR (ddPCR) and LEfSe analysis was used to determine enrichment of infectious taxa at either collection site. Whole genome sequencing and antimicrobial susceptibilities on all BSI isolates.

**Results:**

We collected simultaneous patient stool and blood isolates from 11 *Pseudomonas aeruginosa,* 14 *Escherichia coli*, 4 *Klebsiella sp.,* 8 *Enterococcus sp.,* 18 viridans group streptococci (VGS), 13 *Staphylococcus epidermidis*, and 5 *Staphylococcus aureus* BSIs. Mann-Whitney testing confirmed that individuals with *P. aeruginosa* (oral *P =.004,* stool *P < .001*), *E. coli* (stool *P < .001*), and VGS infections (oral *P =.001*) had significantly higher relative abundance of those respective genera compared to other BSI patients, and this appeared to be site specific. LEfSe analysis confirmed enrichment of *Pseudomonas* in the saliva and stool, *Escherichia* in the stool, and *Streptococcus* in the saliva of respective infections. However, while 64 patients showed presence of the infectious genera in the stool and/or saliva at time of BSI, only 7 exhibited domination. ddPCR was used to confirm not only species specificity, but the detection of those antibiotic resistance determinants (CTX-14, CTX-15, Van A and CfrA) found in the BSI isolates within concurrent stool samples.

**Conclusion:**

It appears that GI domination by the infecting organism was not present at the time of most BSIs. However, in scenarios where the etiological agent was detectable in the saliva or stool, so were the antibiotic resistant determinants. Furthermore, certain infections exhibited a significant difference in oral or stool presence by the same bacteria they were infected with.

**Disclosures:**

**All Authors**: No reported disclosures.

